# The course of problematic social media use in young adolescents: A latent class growth analysis

**DOI:** 10.1111/cdev.13712

**Published:** 2021-11-15

**Authors:** Maartje Boer, Gonneke W. J. M. Stevens, Catrin Finkenauer, Regina J. J. M. van den Eijnden

**Affiliations:** ^1^ Department of Interdisciplinary Social Science Utrecht University Utrecht The Netherlands

## Abstract

Using four waves of longitudinal data collected in 2015–2019 from 1419 Dutch adolescents (*M*_age_ = 12.5, 45.9% female, 21.9% immigrant), this study identified trajectories of problematic social media use (SMU) in parallel with trajectories of SMU frequency. Latent class growth analysis identified two subgroups with relatively high levels of problematic SMU over time: One showed high (24.7%) and one showed average SMU frequency (15.8%). Also, two subgroups with persistently low levels of problematic SMU were identified: One reported low (22.4%) and one reported high SMU frequency (37.1%). Although both subgroups with high levels of problematic SMU reported low subjective well‐being, the group with high SMU frequency showed low self‐control, whereas the group with average SMU frequency reported poor social competencies.

**Abbreviations**
ADHD: attention deficit hyperactivity disorder
BIC: Bayesian information criterion
BLRT: bootstrap likelihood ratio test
DSM‐5: Diagnostic and Statistical Manual of Mental Disorders, 5th ed.
LCGA: latent class growth analysis
LMR‐LRT: Lo–Mendell–Rubin adjusted likelihood ratio test
MLR: maximum likelihood with robust standard errors
SMU: social media use

The current generation of young adolescents grow up in a “hybrid” world, where their offline world is intertwined with online contexts that are facilitated by social media, such as Instagram and Snapchat. Between 2017 and 2019, 63% of 12‐ to 14‐year‐old and 77% of 15‐ and 16‐year‐old European adolescents reported daily usage of social media (Smahel et al., 2020). Other research shows that in 2017 and 2018, a large share of 13‐ and 15‐year‐old European adolescents reported that they were interacting online with friends and others almost all the time throughout the day (36% and 41%, respectively; Inchley et al., 2020). From a developmental perspective, it is understandable why social media are so popular among early and middle adolescents (Granic et al., 2020). Social media allow young adolescents to form and maintain peer relationships (e.g., through instant messaging), to share their perspectives, narratives, and self‐portrayals with others (e.g., by uploading personal photos, videos, and texts), to receive feedback on their appearances and online behaviors (e.g., through “likes” and responses from peers), and to learn from others (e.g., by browsing through peers’ uploads). These functions are all crucial for identity development: a core developmental task of young adolescents (Erikson, 1968).

However, for some adolescents, social media use (SMU) deviates from normative adolescent behavior, namely when they experience symptoms of addiction to social media. In that case, adolescents cannot regulate their SMU: They have social media on top of their mind constantly, feel stress or anxiety when SMU is not possible, or report that their SMU interferes with their functioning in important life domains (Andreassen, 2015; Griffiths et al., 2014). The presence of such addiction symptoms is considered harmful (Griffiths et al., 2014). For instance, meta‐analytic findings indicate that adolescents with such symptoms report low well‐being (Marino et al., 2018). Furthermore, several longitudinal studies, including studies based on data from the present study, suggest that symptoms of addiction toward SMU increase mental health problems, such as depressive symptoms, psychological distress, and attention deficits (Boer, Stevens, Finkenauer, De Looze, et al., 2021; Boer, Stevens, et al., 2020; Chen et al., 2020; Raudsepp, 2019). Nevertheless, social media addiction has not been acknowledged as such in any diagnostic manual, such as the Diagnostic and Statistical Manual of Mental Disorders, 5th ed. (DSM–5; APA, 2013). Therefore, we refer to it as problematic SMU (Lee et al., 2017).

Research in 29 countries showed that in 2017 and 2018, 7% of 11‐ to 15‐year‐olds reported high levels of problematic SMU (Boer, Van den Eijnden, et al., 2020). Despite the growing literature on predictors and outcomes of problematic SMU, studies have not investigated how problematic SMU evolves over time. Consequently, it is unclear whether and for whom problematic SMU persists, increases, or decreases over time. The present study addresses this gap using four annual waves of longitudinal data among Dutch young adolescents. It aims to identify trajectories of problematic SMU and to investigate predictors of these trajectories. Establishing when, to what extent, and among whom problematic SMU emerges identifies windows of opportunity for the development of prevention and intervention programs on problematic SMU. Specifically, it identifies at which period in adolescence the implementation of such programs would be relevant and to whom these programs may be most valuable. Such programs may be important, given the increasing evidence that problematic users face several risks related to their mental health (Boer, Stevens, Finkenauer, De Looze, et al., 2021; Chen et al., 2020; Raudsepp, 2019).

## Trajectories of problematic SMU

To our knowledge, there is currently no theoretical basis and empirical evidence on the course of problematic SMU throughout adolescence, or other behaviors that, similar to problematic SMU, can be characterized as behavioral addictions. As such, hypotheses on how adolescents’ level of problematic SMU develops over time have not yet been advanced. To understand how problematic SMU may evolve, it is important to consider the conceptualization of the behavior: Problematic SMU is characterized by addiction‐like behaviors that are rather exceptional among adolescents (Griffiths, 2013), and can therefore be regarded as deviant behavior. The behavior is conceptually different from (highly) frequent SMU, that is regarded as normative adolescent behavior nowadays. While many adolescents show high SMU frequency (Anderson & Jiang, 2018), this does not necessarily imply loss of control over SMU, which is central to problematic SMU. Furthermore, cross‐sectional as well as longitudinal research shows that problematic SMU is related to lower mental health, while high SMU frequency is not (Boer, Stevens, Finkenauer, De Looze, et al., 2021; Boer, Van den Eijnden, et al., 2020; Shensa et al., 2017). Hence, we pose that problematic SMU reflects deviant behavior that is related to mental health problems.

Therefore, to understand how problematic SMU potentially develops over time, research on adolescents’ developmental trajectories of other deviant behaviors and mental health may provide some directions. Research consistently shows heterogeneous developmental trajectories of, for example, depressive symptoms (Dekker et al., 2007), aggression (Bongers et al., 2004), delinquency (Reinecke, 2006), and binge drinking (Chassin et al., 2002). Together, these studies broadly suggest that adolescents’ vulnerability to problems typically develops through multiple pathways throughout adolescence: One trajectory concerns adolescents who show no or little vulnerability to a specific problem (i.e., persistent low risk), another trajectory concerns adolescents who show relatively persistent high vulnerability to a problem (i.e., persistent high risk), and at least one trajectory concerns adolescents who show variation in problems over time (e.g., temporal, decreasing, or increasing risk). The number and shape(s) of such variable trajectories differ across studies, suggesting that the variability depends on the type of problem investigated. Considering problematic SMU as deviant behavior that is related to low mental health, adolescents’ development of problematic SMU may parallel these broad patterns of trajectories, including a more persistent low‐ and high risk, and one or multiple variable trajectories. Given the possible detrimental impact of problematic SMU (e.g., Chen et al., 2020; Raudsepp, 2019), it is particularly important to investigate whether and which adolescents experience high levels of problematic SMU persistently and thus experience prolonged risks to their mental health throughout their development.

So far, only large‐scale cross‐sectional studies reporting on the average association between age and problematic SMU shed some light on the course of problematic SMU. While some studies show that problematic SMU was more prevalent among older youth (Boer, Van den Eijnden, et al., 2020; Müller et al., 2016), other studies suggest that this was more prevalent among younger youth (Mérelle et al., 2017; Wartberg et al., 2020), and others show no age differences (Bányai et al., 2017; Ho et al., 2017). A possible explanation for these inconclusive findings is that there are subgroups of adolescents with different trajectories of problematic SMU, and that these subgroups were unevenly represented in the samples of previous studies.

## Predictors of problematic SMU

It has been proposed that adolescents with low *subjective well*‐*being* and poor *social competencies*, such as low life satisfaction, low self‐esteem, and poor competencies to form and maintain friendships, are sensitive to problematic SMU. SMU may be especially appealing for adolescents with these psychosocial vulnerabilities, because other than in offline encounters, online they can easily present themselves in a positive way. Consequently, they may develop a preference for online interaction over face‐to‐face and maladaptive cognitions about social media, such as the perception to only have a meaningful life on social media, which may lead to problematic SMU (Caplan, 2003; Davis, 2001). In addition, adolescents with low *self*‐*control*, indicated by attention deficits or impulsivity, have limited ability to inhibit immediate impulses. Therefore, they may not be able to resist temptations and to regulate their SMU, which may make them sensitive to problematic SMU (Mérelle et al., 2017; Wu et al., 2013). However, these propositions lack a developmental perspective, because they do not describe how these psychosocial factors relate to *trajectories* of problematic SMU. That is, whether they increase the risk of, for example, persistently or temporarily high levels of problematic SMU. Identifying which psychosocial profiles increase the risk of following specific trajectories of problematic SMU may support the development of intervention and prevention programs aimed at problematic SMU that target adolescents’ vulnerabilities. These programs are considered particularly relevant for those youth whose high levels of problematic SMU do not desist.

Longitudinal research, under which studies that used data from the present study, examined associations between some of the abovementioned psychosocial factors and problematic SMU (Boer, Stevens, Finkenauer, De Looze, & Van den Eijnden, 2021; Boer, Stevens, et al., 2020; Du et al., 2021; Li et al., 2018). Although these studies provided insight into the average association between psychosocial characteristics and problematic SMU over time, they did not explore whether these factors predict distinct trajectories of problematic SMU. For example, low subjective well‐being may underlie specific trajectories of problematic SMU, but not others. Furthermore, these studies typically focused on predictors of *changes* in problematic SMU, which do not allow for establishing predictors of persistent levels of problematic SMU.

## Current study

Social media are ubiquitous in the daily lives of contemporary adolescents and likely play a significant role in the individual development of particularly young adolescents. Such a context, where social media are omnipresent, may make some adolescents susceptible to developing problematic SMU, which are characterized by symptoms of addiction. Using four waves of longitudinal data with yearly time intervals among Dutch adolescents in early and middle adolescence (*M*_age_ = 12.511, *SD*_age_ = 0.602 in the first wave), this study firstly aimed to explore how adolescents’ level of problematic SMU evolved over time. Based on prior studies on the development of various types of problems during adolescence, we expected to find a persistent low‐ and a high‐risk trajectory, and at least one more variable trajectory of problematic SMU. To consolidate our suggestion that problematic SMU differs from the frequency of SMU and illuminate the similarities or differences between their trajectories, we investigated adolescents’ trajectories of problematic SMU in parallel with their trajectories of SMU frequency. The second aim was to investigate to what extent subjective well‐being (life satisfaction and self‐esteem), low self‐control (attention deficit and impulsivity), and social competencies predicted the identified trajectories. Although research showed that these psychosocial characteristics are related to problematic SMU, their role in particular developments of problematic SMU remains unexplored. Therefore, we did not establish a priori expectations regarding their predictive role in specific trajectories. Thus, given the data‐driven approach to identify trajectories of problematic SMU and its predictors, the design of the present study was considered exploratory.

## METHODS

### Sample

Data came from the Digital Youth project: A longitudinal study among students assessing self‐report Internet‐related behaviors and well‐being (Van den Eijnden et al., 2018). Students were recruited through schools in urban and suburban areas in the Netherlands. Schools were selected based on the project initiator's personal network of contacts with key persons in schools. The data include five waves of data with yearly time intervals, conducted in February–April of 2015 until 2019. In each survey round, students from previous round(s) were invited to participate, but also new students from different grades entered. For the present study, we selected four waves of data from students enrolled in seventh grade at time of the 2015 or 2016 survey rounds, which yielded two subsets: students sampled from 2015 to 2018 (*n* = 1352) and students sampled from 2016 to 2019 (*n* = 998). The two subsets were merged, such that each subset consisted of four waves that we refer to as T1–T4. Hence, growth was modeled as a function of students’ grade, whereby all students were enrolled in seventh grade at T1 and in tenth grade at T4. Students who repeated a class (*n* = 46) or who participated in less than two waves (*n* = 885) were excluded, yielding an analysis sample of 1419 included students. Excluded students reported higher levels of problematic SMU, lower life satisfaction, higher impulsivity, and poorer social competencies than included students, but with small effect sizes (Cohen's *D* = .114–.216). Also, the proportion of boys, adolescents attending pre‐vocational education, and adolescents with an immigrant background was higher among the sample of excluded students, although these differences were very small (Cramer's *V* = .064–.109).

There were few differences between the two subsets from the analysis sample: Adolescents in the second subset reported higher levels of problematic SMU, but also higher life satisfaction than adolescents in the first subset, although these differences were small (Cohen's *D* = .171 and .131, respectively). Additionally, the proportion of adolescents attending pre‐vocational education was highest in the second subset, although here too, the difference was small (Cramer's *V* = .137). Despite these small differences, we found that the (variances of the) initial level and development of problematic SMU and SMU frequency did not vary across the two subsets, suggesting that the distributions of the trajectories were comparable (Table S1). Hence, merging the two samples was justified.

Within the analysis sample, students were on average 12.511 years old (*SD* = 0.602) in T1, 45.9% was girl, and 21.9% had an immigrant background. Among adolescents with an immigrant background, 45.2% had one parent that was born in Suriname, Netherlands Antilles, Morocco, Turkey, or another country, and their other parent was born in the Netherlands. The other 54.8% had two parents from these or other countries. In addition, students followed different educational tracks according to the Dutch education system, namely pre‐vocational (*VMBO*; 57.8%), intermediate (*HAVO*; 28.5%), and pre‐university (*VWO*; 13.7%). The present sample composition differed somewhat from the Dutch school population with respect to educational level: 13.7% among sample participants versus 20.6% in the Dutch 10th‐grade population in 2018 and 2019 (CBS, 2021).

Participation rates at T1 until T4 were 55.1%, 93.5%, 75.9%, and 34.9%, respectively. The reason why data were not complete in T1 was because some students’ first participation was in T2 or T3. Nevertheless, all students were enrolled in the same grade at each assessment. There was considerable dropout among students attending pre‐vocational education: Of all adolescents participating in T4, 19.6% was pre‐vocational student, while of all adolescents participating in T1, this was 60.6%. This dropout was mainly due to dropout of entire pre‐vocational schools, school years (e.g., final exam years), or school classes (e.g., because teachers were not able to schedule the survey assessment), and not due to individual selection.

Prior to each survey assessment, parents received a letter which informed them about the study and provided them with the opportunity to refuse participation of their child via email or telephone call. Also, prior to each survey round, students were informed about the purpose of the study, that participation was voluntary and anonymous, and that they could withdraw their participation at any moment. Both parents and students received this information 2 weeks before the first day of data collection. The assessments were administered in the classroom through digital self‐completion, whereby research‐assistant monitored and assisted students where necessary. The assessments were carried out in accordance with the Declaration of Helsinki and the study procedure was approved by the board of ethics of Utrecht University (FETC16‐076 Eijnden).

### Measures

#### Problematic SMU

**FIGURE 1:**
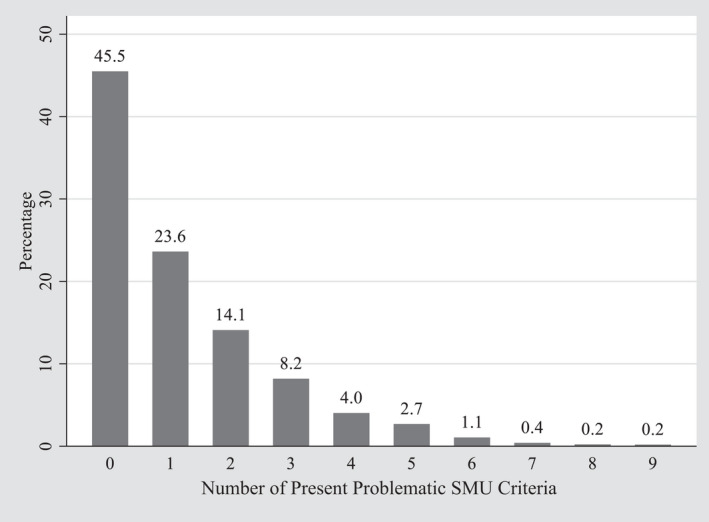
Distribution of problematic SMU. *Note*: The distribution was derived from the complete data on problematic SMU with the data in long format (*n* = 3675 out of 5676 observations). SMU, social media use

We used the nine‐item Social Media Disorder Scale to measure problematic SMU, that measures nine symptoms of addiction to social media, including preoccupation, withdrawal, tolerance, persistence, displacement, conflict, deception, escape, and problems (Van den Eijnden et al., 2016). Respondents were asked, for example, whether in the past year they regularly could not think of anything else but the moment to use social media again (i.e., preoccupation), with a dichotomous response scale (1 *yes* or 0 *no*). The scale corresponds to the nine diagnostic criteria for Internet gaming disorder according to the appendix of the DSM–5, which also follow a dichotomous response structure (APA, 2013; Lemmens et al., 2015). A sum‐score was computed that denotes the number of present criteria. Higher sum‐scores are thereby interpreted as higher levels of problematic SMU. This sum‐score followed a Poisson distribution (Figure 1), corresponding to the distribution observed in a nationally representative sample of 6266 Dutch students aged 12–16 (Boer, Stevens, Finkenauer, Koning, et al., 2021). Thus, high levels of problematic SMU are rather exceptional in the adolescent population, given that most adolescents do not report any problems, whereas a small minority report many. The scale has been found to provide appropriate criterion validity: The higher the level of problematic SMU, the higher the probability of reporting problems related to mental health, school, and sleep, whereby moderate levels of problematic SMU (i.e., endorsement of two to five problematic SMU criteria) are already indicative of a higher risk of problems (Boer, Stevens, Finkenauer, Koning, et al., 2021). As appropriate for dichotomous variables, reliability was calculated using the tetrachoric correlation matrix (Gadermann et al., 2012), yielding an ordinal *α* ranging from .834 to .856 at all waves.

#### SMU frequency

Four items assessed respondents’ SMU frequency (Boer, Stevens, et al., 2020). Respondents were asked how many times per *day* they viewed, per *week* they “liked,” and per *week* they responded to messages, photos, or videos of others on social network sites, such as Facebook, Twitter, Instagram, Google+, or Pinterest (1 *never or less than once* to 7 *more than 40 times*). Respondents were also asked how many times per *day* they send a message, photo, or video via their smartphone via, for example WhatsApp, Chat, SnapChat, or SMS (1 *less than once* to 7 *more than 80 times*). Scores on the four items were averaged, such that the score denoted respondents’ mean level of SMU frequency. Cronbach's *α* ranged between .781 and .853 across all waves. The original scale includes additional items on the frequency of posting a message, photo, or video on social network sites and checking the smartphone for incoming messages, photos, or videos. Of these two items, the first was excluded because it had a low factor loading (<.500) and the second because removal yielded substantial model fit improvement due to high overlap with the other item on smartphone use (*r* = .674–.709).

#### Subjective well‐being

The first indicator of subjective well‐being was *life satisfaction*, using the seven‐item Student's Life Satisfaction Scale (Huebner, 1991). Respondents were asked about their thoughts around their own life, for example, whether they think that their life is going well (1 *strongly disagree* to 6 *strongly agree*). The second indicator was *self*‐*esteem*, using five items of the Rosenberg Self‐esteem Scale (Rosenberg, 1965). Respondents were asked, for example, whether they felt that they have a number of good qualities (1 *strongly disagree* to 5 *strongly agree*). Both scales have been validated among adolescents extensively and adopted in translated form in many adolescent surveys worldwide (Butler & Gasson, 2005; Proctor et al., 2009). For both subjective well‐being indicators, we computed the mean scores of the items. Across all waves, Cronbach's *α* for life satisfaction and self‐esteem ranged from .809 to .838 and from .777 to .825, respectively.

#### Self‐control

The first indicator of self‐control was *attention deficit*, measured with a nine‐item subscale from the attention deficit hyperactivity disorder (ADHD)‐Questionnaire (Scholte & Van der Ploeg, 1999). Respondents were asked, for example, how often they experience difficulties in sustaining prolonged attention on tasks or activities (1 *never* to 5 *very often*). The second indicator was *impulsivity*, measured with a six‐item subscale from the ADHD‐Questionnaire. Respondents were asked, for example, how often they find it difficult to wait for their turn (1 *never* to 5 *very often*). The ADHD‐Questionnaire has been shown to be a reliable and valid measure of ADHD in Dutch adolescents (Scholte & Van der Ploeg, 1999). For both attention deficit and impulsivity, we calculated mean scores using the subscale items. Cronbach's *α* for attention deficit and impulsivity ranged from .860 to .882 and .786 to .834, respectively.

#### Social competencies

We used *perceived friendship competence* as an indicator for social competencies, measured with the five‐item “close friendship”‐subscale of the Self‐Perception Profile for Adolescents (Harter, 2012; Straathof & Treffers, 1989). The subscale has been shown to provide reliable test scores in several adolescent populations (Rose et al., 2012). We used a modified Dutch version of the subscale (Straathof & Treffers, 1989), whereby respondents were asked, for example, whether they find it difficult to form friendships to which they can count on (1 *strongly disagree* to 5 *strongly agree*). Scores were recoded such that high values indicate high levels of social competencies, after which mean scores were computed. Cronbach's *α* ranged from .600 to .709.

#### Controls

The analyses controlled for several time‐invariant characteristics, including *gender* (*boy* or *girl*), *educational level* (*pre*‐*vocational*, *intermediate*, or *pre*‐*university*), and *immigrant background* (*immigrant* or *non*‐*immigrant*). Educational level was determined based on the respondents’ most recent reported level of education. The immigrant background was established based on the country of origin of the respondents’ parent(s), whereby response options were *Netherlands*, *Suriname*, *Netherlands Antilles*, *Morocco*, *Turkey*, and *other countries*. These countries were selected because a large share of the immigrant population in the Netherlands comes from these countries due to colonial past and a history of labor migration to the Netherlands. Adolescents with at least one parent from a different country than the Netherlands were defined as adolescents with an immigrant background.

### Analytic approach

#### Identifying trajectories

We adopted latent class growth analysis (LCGA) using Mplus 8.6 (Muthén & Muthén, 2017). LCGA explores heterogeneity of growth trajectories within a population by classifying individuals into subgroups based on their response patterns (Jung & Wickrama, 2008). It tests several class solutions, whereby each class represents a growth trajectory indicated by an intercept, slope, and quadratic term estimated from multiple repeated measures. Respectively, these three growth parameters denote the average level of problematic SMU at T1, the average change over time, and whether there is nonlinear change. In LCGA‐models, the (co‐)variances of the growth parameters are constrained to zero, which imposes that individuals within a class have similar growth trajectories.

The problematic SMU sum‐score follows a Poisson distribution (Figure 1), which does not allow for ordinary LCGA (Reinecke, 2006). Therefore, we compared the model fit of Poisson and zero‐inflated Poisson growth models. We present our findings using the more parsimonious Poisson models because zero‐inflation parameters were not significant and from three classes onwards model fits were comparable (Figure S1). The trajectories of problematic SMU were estimated in parallel with trajectories of SMU frequency. These co‐trajectories were estimated without any covariates, which facilitates interpretation (Van de Schoot et al., 2017). The model specifications are available in Supporting Information (Figure S2).

The number of classes was established based on the model fit and classification accuracy (Van de Schoot et al., 2017). Model fit was evaluated based on the Bayesian information criterion (BIC). We used the Lo–Mendell–Rubin adjusted likelihood ratio test (LMR‐LRT) and bootstrap likelihood ratio test (BLRT) to indicate whether a class solution improved model fit compared to a class solution with one class less (*p* < .050). Classification accuracy was evaluated based on the average class membership probability of each class, with values close to 1 indicating good classification. Also, Entropy with values of .700 or higher was considered adequate (Reinecke, 2006). As typical for latent class analysis, the model selection was based on a trade‐off between all of the above‐mentioned criteria (Jung & Wickrama, 2008).

The percentage of missing data on the study variables for this part of the analysis ranged from 6.6% (problematic SMU T2) to 65.8% (SMU frequency T4), which was mostly related to dropout. Little's chi‐square test for missing data was significant (*ꭓ* ^2^(118) = 262.144, *p* < .001), which means that we cannot assume that data were completely missing at random. Consequently, listwise deletion of cases with one or multiple missing values may bias results (Enders & Bandalos, 2001). However, in our analysis, we aimed to limit the bias that is associated with missing data by conducting the LCGA using full information maximum likelihood with robust standard errors (MLR), which retains all 1419 respondents.

#### Predictors of trajectories

Based on the latent class solution from the LCGA, we created a nominal class variable that denotes the most likely class membership for each respondent. In addition, we computed respondents’ average level of subjective well‐being, self‐control, and social competencies using their responses from T1 until T4. These person‐specific means denote the time‐invariant (i.e., trait‐like, stable) part of adolescents’ level of subjective well‐being, self‐control, and social competencies. Subsequently, we conducted a multivariate multinomial regression analysis to predict class membership with the subjective well‐being, self‐control, and social competencies person‐specific means, while controlling for demographic characteristics. In doing so, we specified the measurement error of the class variable using the logits for the classification probabilities for the most likely latent class membership as obtained from the LCGA. This model specification takes into account the uncertainty that is associated with the classification, which improves the accuracy of the multinomial regression estimates (Asparouhov & Muthén, 2014).

There was one missing observation for immigrant background. For the predictors, the percentage of missing data ranged between 6.6% (attention deficit T2) and 65.9% (life satisfaction T4), which was mostly related to dropout. Gender, educational level, and the class variable were complete. The missing data were not found to be completely missing at random (*ꭓ* ^2^(604) = 802.317, *p* < .001), which means that retaining all respondents is required to limit possible bias (Enders & Bandalos, 2001). However, with the present multinomial model, MLR‐estimation did not retain all respondents. Therefore, for the multinomial analysis, we imputed missing values using multiple imputation with chained equations (Royston & White, 2011). In this procedure, missing values were estimated based on predictive mean matching with “five nearest neighbors,” whereby missing values were imputed based on the observed data on the study variables. Imputations were computed in Stata 13.0 (StataCorp, 2013) and exported to Mplus 8.6 (Muthén & Muthén, 2017) to conduct the multinomial analysis.

### Preregistration

The subsample selection and analytical approaches were preregistered. In order to improve the analytical approach, we deviated from the preregistration by defining the predictors of the trajectories using the person‐specific averages across all waves instead of using only the T1 data. Also, the analysis sample yielded 1419 adolescents instead of the preregistered 1414, which was due to a correction on the sample selection. For the remainder, all analyses followed the preregistered procedures. The preregistration, codes for data selection and all analyses, and the Supporting Information may be consulted via https://osf.io/r9t4a/.

## RESULTS

### Descriptive statistics

Table 1 shows the descriptive statistics for all study variables. It shows that the observed average level of problematic SMU was low, whereas the level of SMU frequency was around the midpoint of its scale. Observed averages in life satisfaction, self‐esteem, and social competencies were high, whereas attention deficit and impulsivity were low, given the ranges of the respective scales.

**TABLE 1:** Descriptive statistics and correlations of the study variables (*n* = 1419)

		*T*	*M*/%	*SD*	Min.	Max.	1	2	3	4	5	6	7
							*r*	*r*	*r*	*r*	*r*	*r*	*r*
1	Problematic SMU	1	1.129	1.494	0	8							
		2	1.293	1.616	0	9							
		3	1.152	1.464	0	9							
		4	1.065	1.431	0	9							
2	SMU frequency	1	3.923	1.586	1.000	7.000	.341***						
		2	4.271	1.565	1.000	7.000	.317***						
		3	4.285	1.485	1.000	7.000	.243***						
		4	4.296	1.376	1.000	7.000	.251***						
3	Life satisfaction	1	4.932	0.757	1.143	6.000	−.282***	−.098**					
		2	4.687	0.874	1.000	6.000	−.310***	−.089**					
		3	4.540	0.876	1.000	6.000	−.306***	−.044					
		4	4.476	0.884	1.000	6.000	−.241***	−.028					
4	Self‐esteem	1	3.946	0.685	1.200	5.000	−.198***	−.066	.516***				
		2	3.865	0.758	1.000	5.000	−.270***	−.109***	.628***				
		3	3.757	0.738	1.000	5.000	−.297***	−.052	.628***				
		4	3.730	0.745	1.000	5.000	−.180***	−.032	.593***				
5	Attention deficits	1	2.115	0.703	1.000	4.333	.349***	.193***	−.318***	−.191***			
		2	2.266	0.773	1.000	5.000	.379***	.236***	−.287***	−.246***			
		3	2.399	0.754	1.000	5.000	.312***	.150***	−.257***	−.254***			
		4	2.463	0.783	1.000	5.000	.267***	.108**	−.199***	−.214***			
6	Impulsivity	1	1.863	0.682	1.000	4.667	.357***	.276***	−.170***	−.081**	.656***		
		2	1.926	0.745	1.000	5.000	.357***	.264***	−.201***	−.167***	.683***		
		3	1.947	0.678	1.000	5.000	.308***	.200***	−.178***	−.168***	.624***		
		4	1.934	0.650	1.000	4.000	.241***	.172***	−.085*	−.134***	.642***		
7	Social competence	1	4.401	0.628	1.800	5.000	−.197***	.067	.285***	.291***	−.156***	−.146***	
		2	4.354	0.681	1.200	5.000	−.108***	.117***	.208***	.253***	−.148***	−.166***	
		3	4.322	0.700	1.000	5.000	−.127***	.098**	.303***	.360***	−.185***	−.214***	
		4	4.352	0.675	1.200	5.000	−.137**	.211***	.275***	.308***	−.115**	−.060	
8	Female	1–4	45.9		0	1	−.060	.163***	−.102*	−.169***	−.150**	−.265***	.161**
							.079*	.276***	−.112**	−.148***	−.022	−.164***	.231***
							.084*	.213***	−.032	−.088*	−.073	−.177***	.208***
							.066	.196***	−.053	−.047	−.068	−.117*	.283***
9	Pre‐vocational education	1–4	57.8		0	1	.322***	.237***	−.082	−.015	.135**	.221***	−.097
							.150***	.171***	.010	−.064	.052	.190***	−.035
							.104**	.164***	.012	−.010	−.026	.076*	−.035
							.094*	.099**	−.011	−.096*	−.117**	−.011	−.103**
10	Immigrant background	1–4	21.8		0	1	.076	.046	−.022	−.043	−.077	−.005	.028
							.041	−.024	.102*	.099**	−.106**	−.015	.002
							.017	−.034	<.001	.075	−.066	.048	−.075
							−.019	−.087	−.159*	<.001	−.040	−.064	−.252***

Note

Means and standard deviations are based on complete data.

Abbreviations: *M*, mean; Max., maximum; Min., minimum; *r*, pairwise correlation; *SD*, standard deviation; SMU, social media use; *T*, timepoint (i.e., wave).

* *p* < .05

** *p* < .01

*** *p* < .001.

### Identifying trajectories

#### Average trajectory

**FIGURE 2:**
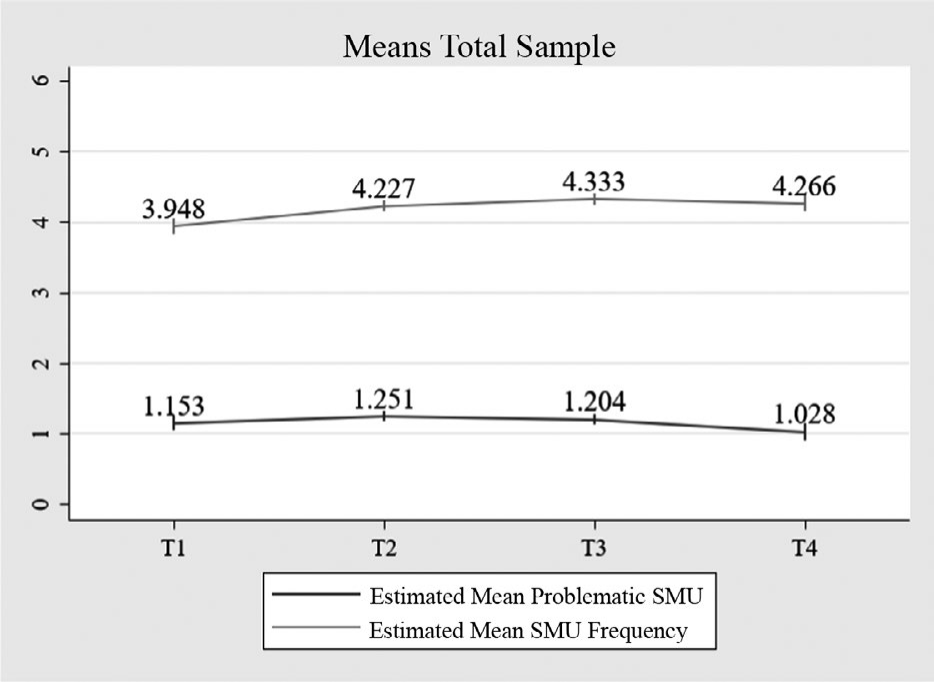
Average co‐trajectory of problematic SMU and SMU frequency, *n* = 1419. *Note*: Data labels show the model estimated means based on the one‐class solution. Vertical bars denote the 95% confidence intervals. SMU, social media use

Figure 2 shows the estimated average trajectory of problematic SMU and SMU frequency over time. At T1, the average reported level of problematic SMU was 1.153. The course of problematic SMU was nonlinear, whereby adolescents’ level of problematic SMU first increased, but decreased after T2 (*B*_linear_ = 0.142, *p* = .015; *B*_quadratic_ = −0.060, *p* = .002). Also, there was a nonlinear trend of SMU frequency, whereby SMU frequency increased until T3, but decreased thereafter (*B*_linear_ = 0.366, *p* < .001; *B*_quadratic_ = −0.087, *p* < .001).

#### Model selection

Table 2 shows the fit indices and classification accuracy of six LCGA models. The higher the number of classes, the better the model fit in terms of the BIC and BLRT, as the BIC decreased until the final model and the BLRT *p*‐value indicated that adding classes improved model fit compared to a model with one class less (*p* < .001). However, for the 5‐ and 6‐class models, the decrease in BIC was relatively small, and for the 6‐class model, the LMR‐LRT *p*‐value was not significant (*p* = .172). Furthermore, the Entropy of the 5‐ and 6‐class models was below .700, which suggests inaccurate classification accuracy. Hence, the 1‐ to 4‐class solutions seemed more eligible. From these models, we selected and further interpreted the 4‐class solution, because this model showed the best model fit according to all fit indices, and the Entropy was appropriate (.719).

**TABLE 2:** Model fit indices and classification accuracy LCGA models (*n* = 1419)

Par.	C	BIC	Entropy	LMR‐LRT value	LMR‐LRT *p*‐value	BLRT *p*‐value	Min. class size	Max. class size	Min. probability	Max. probability
10	1	25,439.225					1419	1419	1	1
17	2	23,588.660	.723	1864.667	<.001	<.001	691	728	.916	.918
24	3	23,081.855	.708	546.846	<.001	<.001	387	578	.847	.891
31	4	22,868.471	.719	259.088	<.001	<.001	224	527	.728	.896
38	5	22,806.901	.673	110.204	.035	<.001	238	350	.748	.874
45	6	22,780.090	.665	76.115	.172	<.001	139	348	.650	.802

Abbreviations: BIC, Bayesian information criterion; BLRT, bootstrap likelihood ratio test; C, number of classes; LCGA, latent class growth analysis; LMR‐LRT, Lo–Mendell–Rubin likelihood ratio test; Max., maximum; Min., minimum; Par., number of free parameters.

#### Interpretation of trajectories

**FIGURE 3:**
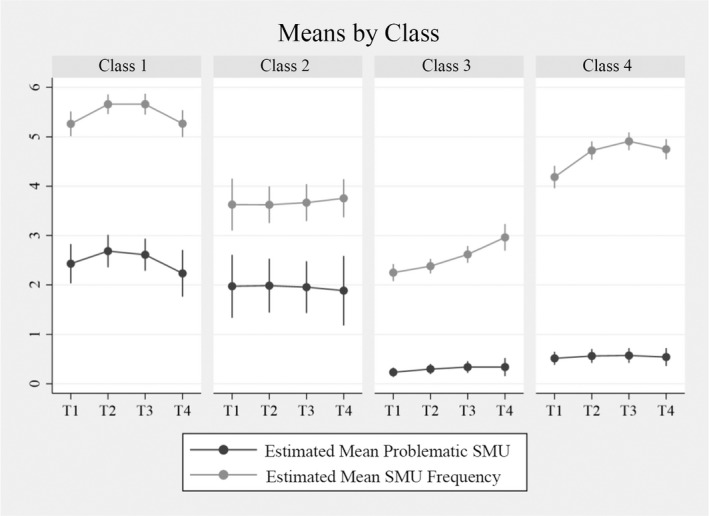
Average co‐trajectory of problematic SMU and SMU frequency by latent class, *n* = 1419. *Note*: Vertical bars denote the 95% confidence intervals. SMU, social media use

Table 3 reports the model estimates and group sizes of the classes from the 4‐class solution and Figure 3 shows the estimated means of problematic SMU and SMU frequency. In the first class, adolescents reported relatively high levels of problematic SMU (*M*_T1_ = 2.430). Their level of problematic SMU followed a nonlinear trend, whereby problematic SMU first increased, but decreased after T2. Adolescents in Class 1 also reported higher levels of SMU frequency than adolescents in the other classes (*M*_T1_ = 5.264). The course of their SMU frequency was nonlinear, whereby they first showed an increase, followed by a decrease. In Class 2, which had the least members (15.8%), the average level of problematic SMU was also relatively high, but stable over time (*M*_T1_ = 1.973). Adolescents within this group, however, reported average levels of SMU frequency and this level remained stable over time (*M*_T1_ = 3.628). In Class 3, adolescents reported the lowest level of problematic SMU, which was stable over time (*M*_T1_ = 0.233). Also, adolescents’ SMU frequency was lower than average, with no significant changes over time (*M*_T1_ = 2.249). In Class 4, which included the most members (37.1%), adolescents’ level of problematic SMU was also lower than average and stable over time (*M*_T1_ = 0.515), but the level of SMU frequency over time was higher than in Class 2 and 3, with a significant nonlinear course: SMU frequency increased, but this increase became smaller over time and eventually decreased in T4 (*M*_T1_ = 4.186).

**TABLE 3:** Model estimates 4‐class solution

	Class 1: *n *= 350 (24.7%) Variably high problematic SMU, variably high SMU frequency			Class 2: *n *= 224 (15.8%) Persistently high problematic SMU, persistently average SMU frequency			Class 3: *n *= 318 (22.4%) Persistently low problematic SMU, persistently low SMU frequency			Class 4: *n *= 527 (37.1%) Persistently low problematic SMU, variably high SMU frequency		
	*B*	*SE*	D.	*B*	*SE*	D.	*B*	*SE*	D.	*B*	*SE*	D.
Problematic SMU												
Intercept	0.888***	.084	a	0.680***	.165	a	−1.458***	.212	b	−0.665***	.131	c
Slope	0.164	.089	a	0.017	.150	a	0.310	.256	a	0.128	.182	a
Quadratic	−0.064*	.031	a	−0.011	.053	a	−0.062	.088	a	−0.037	.060	a
SMU frequency												
Intercept	5.264***	.128	a	3.628***	.269	b	2.249***	.089	c	4.186***	.116	b
Slope	0.592***	.137	ac	−0.027	.294	ab	0.077	.106	b	0.711***	.119	c
Quadratic	−0.197***	.042	a	0.023	.083	b	0.054	.037	b	−0.174***	.036	a

Note

Columns with different letters denote that estimates differed significantly across the respective classes as obtained by *z*‐scores of the parameter differences.

Abbreviations: *B*, unstandardized coefficient; D., difference; *SE*, standard error; SMU, social media use.

* *p *< .05

** *p* < .01

*** *p* < .001.

To gain a more detailed understanding of the classes, we report on some of the class comparisons using the *z*‐scores of the parameter differences (Table 3). The intercept of problematic SMU differed significantly between all classes, except between Classes 1 and 2. This means that except for these two classes, all classes had different levels of problematic SMU at T1. The intercept of SMU frequency differed across all classes, except for Classes 2 and 4. Also, the nonlinear trends of SMU frequency in Classes 1 and 4 were not significantly different.

### Predictors of trajectories

Table 4 displays the differences in adolescents’ demographic characteristics, subjective well‐being, self‐control, and social competencies by class. We examined whether these factors predicted class membership using multivariate multinomial regression. Given that Class 4 had the most members, we conducted the multinomial analysis using Class 4 as the reference group. Hence, estimates from this analysis indicate the extent to which, for example, higher levels of attention deficits, increase the probability of following the trajectories of Classes 1, 2, or 3, relative to Class 4. For reference, additional findings from stepwise analyses can be consulted in the Appendix (Tables A1, A2, A3).

**TABLE 4:** Observed means and proportions study variables, by class (*n* = 1419)

	Pooled sample		Class 1: Variably high problematic SMU, variably high SMU frequency		Class 2: Persistently high problematic SMU, persistently average SMU frequency		Class 3: Persistently low problematic SMU, persistently low SMU frequency		Class 4: Persistently low problematic SMU, variably high SMU frequency	
	*M*/%	*SD*	*M*/%	*SD*	*M*/%	*SD*	*M*/%	*SD*	*M*/%	*SD*
Controls										
Girl	45.9		58.0		35.7		30.8		51.4	
Pre‐vocational education	57.8		70.0		59.8		50.3		53.3	
Intermediate education	28.5		22.0		30.4		29.9		31.3	
Pre‐university education	13.7		8.0		9.8		19.8		15.4	
Immigrant background^a^	21.8		21.4		26.8		22.6		19.5	
Subjective well‐being										
Life satisfaction^a^	4.661	0.595	4.449	0.644	4.500	0.616	4.786	0.558	4.796	0.512
Self‐esteem^a^	3.815	0.491	3.701	0.503	3.668	0.489	3.944	0.474	3.877	0.462
Self‐control										
Attention deficit^a^	2.285	0.526	2.555	0.507	2.383	0.490	2.081	0.501	2.188	0.485
Impulsivity^a^	1.918	0.484	2.204	0.513	1.989	0.447	1.676	0.388	1.843	0.428
Social competencies										
Perceived friendship competence^a^	4.321	0.460	4.324	0.430	4.035	0.510	4.297	0.477	4.454	0.383

Abbreviations: *M*, mean; *SD*, standard deviation; SMU, social media use.

^a^ Proportion, means, and standard deviations for the pooled sample slightly differ from those reported in the sample description and Table 1. This is because the present table presents the proportions, means, and standard deviations based on the imputed data, whereas Table 1 presents the proportions, means and standard deviations based on the complete data.

#### Class 1: Variably high problematic SMU, variably high SMU frequency

**FIGURE 4:**
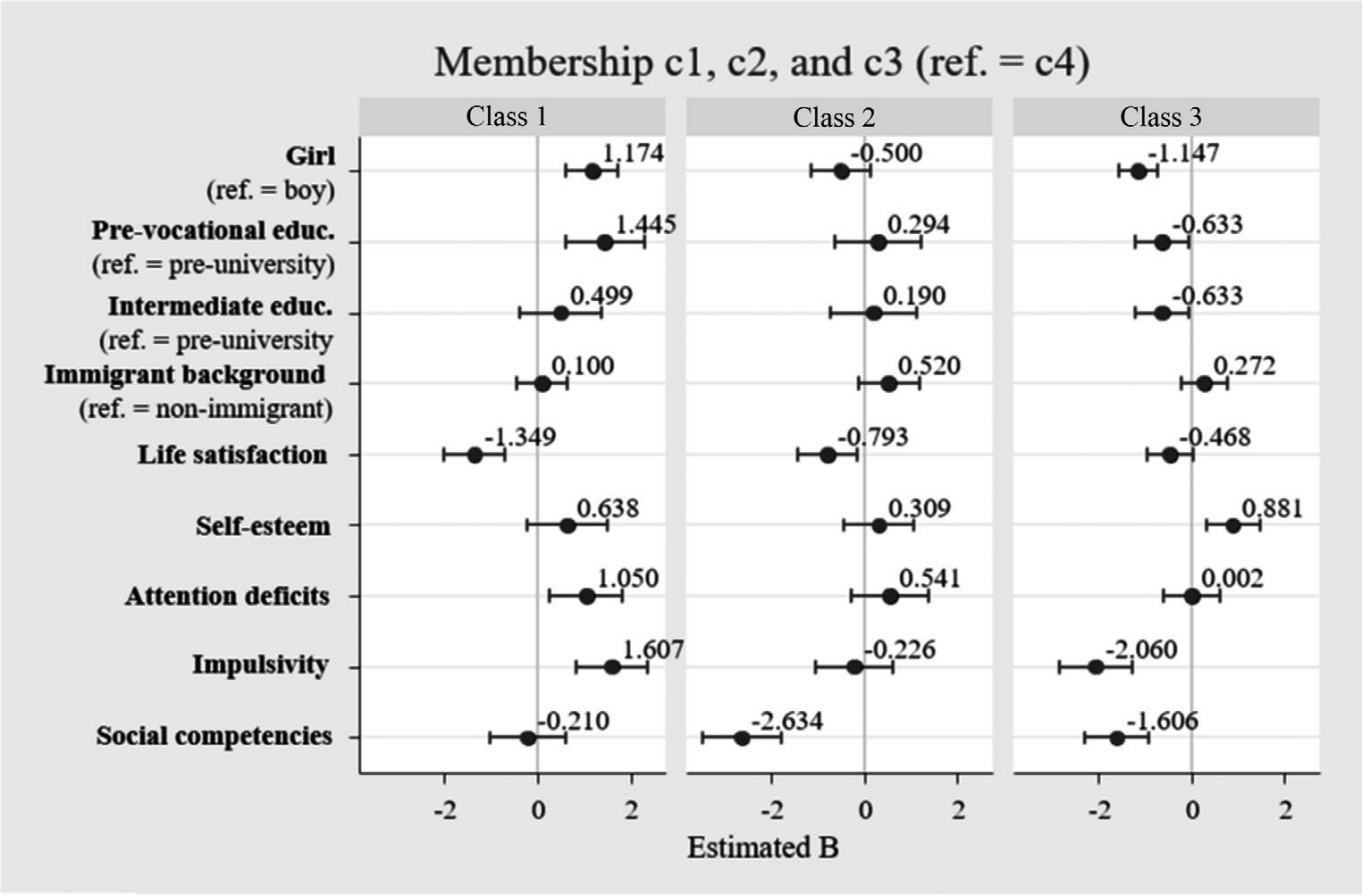
Estimates from multivariate multinomial regression analysis on class membership, *n* = 1419. *Note*: Estimates are logit coefficients. Ref. = reference category. Class 1 = variably high problematic SMU, variably high SMU frequency; Class 2 = persistently high problematic SMU, persistently average SMU frequency; Class 3 = persistently low problematic SMU, persistently low SMU frequency; Class 4 = persistently low problematic SMU, variably high SMU frequency. SMU, social media use

Figure 4 shows that compared to Class 4, girls and pre‐vocational educated adolescents were more likely to be in Class 1 than boys and pre‐university adolescents, respectively (*B* = 1.174, *p* < .001, OR = 3.241 and *B* = 1.445, *p* = .001, OR = 4.250). Lower life satisfaction (*B* = −1.349, *p* < .001, OR = 0.263), higher attention deficit (*B* = 1.050, *p* = .008, OR = 2.914), and higher impulsivity (*B* = 1.607, *p* < .001, OR = 5.051), were associated with a greater probability of being in Class 1 compared to Class 4.

#### Class 2: Persistently high problematic SMU, persistently average SMU frequency

Class 2 and 4 did not vary by demographic characteristics. The lower the level of life satisfaction and social competencies, the higher the probability of being in Class 2 compared to Class 4 (*B* = −0.793, *p* = .013, OR = 0.454; *B* = −2.634, *p* < .001, OR = 0.073).

#### Class 3: Persistently low problematic SMU, persistently low SMU frequency

Relative to Class 4, boys were more likely to be in Class 3 than girls (*B* = −1.147 *p* < .001, OR = 0.318). Also, adolescents attending pre‐university education had a higher probability of being in Class 3 than adolescents attending intermediate or pre‐vocational education (*B* = −0.633, *p* = .028, OR = 0.532; *B* = −0.633, *p* = .031, OR = 0.531). Higher self‐esteem (*B* = 0.881, *p* = .003, OR = 2.423), lower impulsivity (*B* = −2.060, *p* < .001, OR = 0.130), and poorer social competencies (*B* = −1.606, *p* < .001, OR = 0.202) were associated with a greater probability of being in Class 3.

#### Additional class comparisons

We also explored other class differences by repeating the multivariate multinomial analysis with other reference categories (see Appendix, Table A4). This analysis was not preregistered and therefore considered as additional exploratory analysis. Comparing the classes with the highest level of problematic SMU (Classes 1 and 2), results showed that levels of impulsivity and social competence were higher in Class 1 than in Class 2 (*B* = 1.833, *p* < .001, OR = 6.327; *B* = 2.424, *p* < .001, OR = 11.536). In addition, adolescents in Class 1 showed lower life satisfaction (*B* = −0.881, *p* = .020, OR = 0.423), higher attention deficit and impulsivity (*B* = 1.048, *p* = .017, OR = 2.928; *B* = 3.667, *p* < .001, OR = 41.106), and stronger social competencies (*B* = 1.369, *p* = .005, OR = 4.182), than adolescents in Class 3. Adolescents in Class 2 showed higher impulsivity and weaker social competencies than adolescents in Class 3 (*B* = 1.834, *p* < .001, OR = 6.392; *B* = −1.028, *p* = .014, OR = 0.365).

## DISCUSSION

The present study investigated adolescents’ trajectories of problematic SMU in parallel with their trajectories of SMU frequency in early and middle adolescence. Four subgroups were identified: Two subgroups that showed relatively high levels of problematic SMU over time, of which one reported high and one reported average levels of SMU frequency, and two subgroups that showed low levels of problematic SMU over time, of which one reported low and one reported high levels of SMU frequency. In the subgroup with relatively high levels of problematic SMU and SMU frequency, problematic SMU first increased, but decreased after the second year, although the level of problematic SMU remained high. In the other three subgroups, the levels of problematic SMU were persistent over time. The subgroup with low levels of problematic SMU but high SMU frequency had the most members. Relative to this group, adolescents in the two subgroups with high levels of problematic SMU showed the most problematic profiles regarding their psychosocial characteristics, although the profiles of these two subgroups differed. Particularly, although both subgroups showed lower levels of subjective well‐being (i.e., lower life satisfaction), the subgroup with high levels of problematic SMU and SMU frequency showed lower levels of self‐control (i.e., higher attention deficit and impulsivity), whereas the subgroup with high levels of problematic SMU and average SMU frequency reported poorer social competencies (i.e., perceived friendship competencies).

In line with studies investigating developmental trajectories of mental health problems and deviant behaviors throughout adolescence, such as depressive symptoms, aggression, delinquency, or binge drinking (Bongers et al., 2004; Chassin et al., 2002; Dekker et al., 2007; Reinecke, 2006), problematic SMU evolved through persistently high, persistently low, and variable trajectories. For both subgroups with relatively high levels of problematic SMU, the level of problematic SMU remained high throughout the entire 4‐year period, implying that high levels of problematic SMU are rather persistent over time. This finding is plausible, given the addiction‐like nature of problematic SMU. Characteristic for behavioral addiction is that it is difficult to resist the temptation to engage in the behavior or to reduce it, and that it persists over a significant period of time (Kardefelt‐Winther et al., 2017). Also, it is conceivably challenging for adolescents with higher levels of problematic SMU to regain control over their SMU, given that they can access social media on their smartphones anytime wherever they are. Furthermore, nowadays, abstaining from social media may be difficult for young adolescents, given that many activities that are relevant to their social and educational development take place online. For example, abstaining may come at the expense of social connection with peers or schoolwork. These functions of social media may make it almost impossible to resist the temptation and impulse to use social media, and thus to overcome problematic SMU.

Notwithstanding the finding that higher levels of problematic SMU remained high, there may still be adolescents with more variable trajectories of problematic SMU. After all, the present study investigated *average* subgroup trajectories, whereas there may be individual differences in trajectories and their development. Furthermore, the course of adolescents’ problematic SMU may change when they enter late adolescence, which was not measured by the present study. For example, research shows that for some subgroups of adolescents, problem behaviors, such as depressive symptoms and binge drinking, may increase or decrease during late adolescence (Chassin et al., 2002; Dekker et al., 2007).

Although there were two groups with relatively high levels of problematic SMU compared to the average level in the sample and reported in other research (Boer, Stevens, Finkenauer, Koning, et al., 2021), the absolute levels of problematic SMU within these two groups were rather moderate. Nevertheless, moderate levels of problematic SMU may already threaten important life domains, as cross‐level research shows that endorsing moderate levels of problematic SMU is associated with a high risk of, for example, reporting schoolwork pressure and poor sleep quality (Boer, Stevens, Finkenauer, Koning, et al., 2021). However, longitudinal research is required to establish whether moderate levels of problematic problems indeed increase such problems over time. This research is considered important, because if moderate levels of problematic SMU are harmful to young adolescents, then this highlights the importance of prevention and intervention programs at schools aimed at decreasing adolescents’ (risk of developing) problematic SMU. After all, our findings suggest that a substantial group of young adolescents experience such levels of problematic SMU for a prolonged period of time.

Another important finding was that the four identified subgroups showed different co‐developments of problematic SMU and SMU frequency. For example, in the largest subgroup, adolescents reported persistently low levels of problematic SMU with variably high levels of SMU frequency. Another subgroup, though relatively small, showed high levels of problematic SMU with average SMU frequency, which suggests that some adolescents endorse problematic SMU without using social media intensively. Overall, the finding that trajectories of SMU frequency do not necessarily parallel trajectories of problematic SMU supports the proposition that problematic SMU and SMU frequency should be considered as different dimensions related to SMU (Boer, Van den Eijnden, et al., 2020). This finding is in line with large‐scale and case studies on gaming, which show that excessive gaming does not necessarily imply problematic gaming (Griffiths, 2010; Király et al., 2017). Furthermore, the finding that the subgroup with persistently low levels of problematic SMU and variably high SMU frequency had the most members informs parents, teachers, and policymakers who are concerned about adolescents’ SMU that it is rather normative that adolescents display high SMU frequency and that this does not necessarily imply experiencing problematic SMU. Thus, rather than problematizing high SMU frequency, it is important to recognize that it is often common behavior for today's adolescents instead of a risk factor for problematic SMU.

The two subgroups with relatively high levels of problematic SMU showed different profiles. In one subgroup, adolescents reported high levels of SMU frequency, were more often female, more often followed pre‐vocational education, and reported low subjective well‐being and self‐control, which is in line with previously found predictors of problematic SMU (Bányai et al., 2017; Mérelle et al., 2017). The profile of the other subgroup was less typical, because adolescents in this group showed average levels of SMU frequency. This latter group also showed lower subjective well‐being, but in addition, reported poorer social competencies. One possible explanation for the finding that adolescents with poor social competencies and high levels of problematic SMU reported average SMU frequency may be a mismatch between their desired and actual social network size. Due to their lack of social competencies, they may not have the social network they desire. Consequently, they may become preoccupied with the social media activities of others, without having the desired social network to actively interact with online. Overall, these findings confirm that psychosocial vulnerabilities, including poor subjective well‐being, low self‐control, and low social competence, are linked to problematic SMU (Caplan, 2003; Davis, 2001; Mérelle et al., 2017), but they also extend the literature in two ways. First, these characteristics increase the risk of experiencing *persistently* higher levels of problematic SMU during early and middle adolescence, which implies that vulnerable adolescents face prolonged sensitivity to problematic SMU throughout this period. Second, problematic SMU manifests in different ways, depending on the risk profile: While problematic SMU of adolescents with low self‐control seems externally visible through high SMU frequency, problematic SMU of adolescents with low social competencies may be more internally present, given that they do not show high SMU frequency. This finding suggests that for this latter group, problematic SMU may be more difficult to detect for professionals and parents that are concerned with the well‐being of young adolescents.

In addition, we did not observe a variable trajectory that captured the onset of problematic SMU, which implies that problematic SMU may emerge more at the start of early adolescence. Correspondingly, research shows that 11‐year‐olds may already endorse multiple problematic SMU criteria (Stevens et al., 2018). Other research among Dutch children shows that in 2017, the percentage of 10‐year‐olds that used Whatsapp, Snapchat, and Instagram was 69%, 25%, and 19%, respectively. Among 11‐year‐olds, this was 82%, 30%, and 35%, respectively (Kennisnet, 2017). Given that the majority of the 10‐ and 11‐year‐olds use social media and that they may already experience problematic SMU in this period, it is important that parents and teachers monitor and support children around this age who experience severe problems in regulating their use or whose use goes at the expense of activities important to children's health.

This study showed that lower subjective well‐being and self‐control predicted problematic SMU, although this was not found in previous research using the same data (Boer, Stevens, Finkenauer, De Looze, et al., 2021; Boer, Stevens, et al., 2020). It should be noted, however, that previous research focused on within‐person processes and showed that adolescents with lower levels of subjective well‐being or self‐control *relative to their individual average* did not show an *increase in problematic SMU*. The present study focused on between‐person comparisons and showed that adolescents with lower levels of subjective well‐being and self‐control *relative to other adolescents* were likely to report *persistent and variable high levels of problematic SMU*. Together, this suggests that particularly the between‐person (i.e., trait‐like) differences in well‐being and self‐control may determine adolescents’ vulnerability to problematic SMU.

### Limitations and future directions

The findings of the present study should be interpreted in light of several limitations. First, we investigated adolescents’ trajectories during a limited time span, that is, 4 years within early and middle adolescence. During this period, social media may play a larger role in adolescents’ daily lives than in other periods, because this period typically revolves around forming new friendships, exploring new perspectives, and constructing and sharing personal narratives, which can be facilitated by social media. When these developmental tasks are (partly) fulfilled and personal needs change, different trajectories may emerge. As such, we expect that findings from the present study cannot be generalized to older adolescents. Future research comparing trajectories of problematic SMU across younger and older adolescents would improve our understanding of adolescents’ problematic SMU in the context of their developmental period. Second, the present study only assessed trajectories of Dutch adolescents. There are substantial cross‐national differences in young adolescents’ level of problematic SMU and within the European region, high levels of problematic SMU are the least prevalent in the Netherlands (Boer, Van den Eijnden, et al., 2020). As such, trajectories of Dutch adolescents may deviate from trajectories of adolescents from other cultures. Third, we used self‐report measures to indicate SMU frequency, which may have limited accuracy (Junco, 2013). Researchers stressed that the frequency of SMU may be difficult to recall and to estimate (Parry et al., 2020), which is plausible given that SMU typically occurs fragmented throughout the entire day. More objective assessments would be necessary to diminish the influence of recall biases, which furthermore also reduce socially desirable responding biases. Hence, to gain more insight into the co‐trajectory of adolescents’ SMU frequency and problematic SMU, replicating our study using more objective measures of SMU, such as time‐tracking applications, are considered promising. Fourth, we determined the time‐invariant (i.e., trait‐like, stable) part of adolescents’ level of subjective well‐being, self‐control, and social competencies based on four waves of data across 4 years. However, a longer time frame may facilitate more accurate estimates of trait‐like psychosocial factors. Therefore, to gain more robust insights into the explanatory role of the investigated psychosocial characteristics in adolescents’ trajectories of problematic SMU, more research using longitudinal data across a longer time span (e.g., from middle childhood to late adolescence) is considered important. Fifth, because participating schools were not sampled through a random sampling selection procedure, the generalizability of our findings to the young adolescent population may be limited. Sixth, the present study dealt with considerable amounts of missing data. Although we aimed to limit any potential bias related to missing data by applying modern missing data techniques including full information maximum likelihood and multiple imputation instead of, for example, listwise deletion (Enders & Bandalos, 2001; Peeters et al., 2015), we acknowledge that we cannot exclude the possibility that the missing data affected the estimates of the present analyses. Considering the fifth and sixth limitation, prospective longitudinal studies on trajectories of problematic SMU using more representative and complete samples are warranted. Seventh, the present study measured one particular social competence, namely perceived friendship competencies. Future studies on other social competencies in relation to trajectories of problematic SMU may enhance current knowledge on the role of young adolescents’ social competencies in developing problematic SMU. In doing so, focusing on peer reputation is considered promising, given that young adolescents often perceive this as highly important (LaFontana & Cillessen, 2010).

## CONCLUSION

Given the increasing evidence suggesting that problematic SMU hampers young adolescents’ well‐being, it is important to identify who develops problematic SMU and how it develops during adolescence. The present study is a first step to identify trajectories of problematic SMU among young adolescents and thereby uniquely contributes toward understanding the course of problematic SMU. We identified two subgroups of adolescents who showed relatively high levels of problematic SMU that remained high over time, which suggests that problematic SMU is likely to persist. Adolescents in these two subgroups showed different profiles: One subgroup was characterized by high SMU frequency over time, low life satisfaction, and low self‐control, whereas the other subgroup was characterized by average SMU frequency over time, low life satisfaction, and poorer social competencies. Developing prevention and intervention programs on (reducing levels of) problematic SMU may be important, given the persistent nature of problematic SMU among young adolescents. Such programs may target adolescents’ psychosocial vulnerabilities that possibly play a role in developing problematic SMU. Notwithstanding these findings, most adolescents endorsed persistently low levels of problematic SMU with high SMU frequency, suggesting that high SMU frequency is normative and not necessarily a risk factor for developing problematic SMU.

## CONFLICT OF INTEREST

The authors of this manuscript do not have any conflicts to declare.

## Supporting information

Supplementary Material

## Appendix

**TABLE A1:** Results multinomial regression, membership class 1 (*n* = 1419)

	Class 1: Variably high problematic SMU, variably high SMU frequency (ref. = class 4: persistently low problematic SMU, variably high SMU frequency)														
	Model A			Model B			Model C			Model D			Model E (Figure 4)		
	*B*	*SE*	*OR*	*B*	*SE*	*OR*	*B*	*SE*	*OR*	*B*	*SE*	*OR*	*B*	*SE*	*OR*
Controls															
Girl (ref. = boy)	0.445*	.192	1.560	0.376	.213	1.457	1.190***	.257	3.288	0.643**	.203	1.903	1.174***	.289	3.241
Pre‐vocational education (ref. = pre‐university)	1.319***	.340	3.741	1.256***	.337	3.511	1.501**	.439	4.489	1.259***	.350	3.523	1.445**	.433	4.250
Intermediate education (ref. = pre‐university)	0.482	.364	1.619	0.314	.360	1.369	0.696	.464	2.006	0.453	.372	1.573	0.499	.455	1.649
Immigrant background (ref. = non‐immigrant)	−0.002	.225	0.998	0.071	.239	1.074	0.112	.264	1.120	−0.045	.233	0.956	0.100	.279	1.108
Subjective well‐being															
Life satisfaction				−1.530***	.298	0.218							−1.349***	.331	0.263
Self‐esteem				0.098	.331	1.115							0.638	.441	1.940
Self‐control															
Attention deficit							1.341***	.356	3.875				1.050**	.399	2.914
Impulsivity							1.599***	.338	4.972				1.607***	.393	5.051
Social competencies															
Perceived friendship competence										−1.296***	.328	0.276	−0.210	.415	0.824

Abbreviations: *B*, logit coefficient; *OR*, odds ratio; Ref., reference category; *SE*, standard error; SMU, social media use.

* *p* < .05

** *p* < .01

*** *p* < .001.

**TABLE A2:** Results multinomial regression, membership class 2 (*n* = 1419)

	Class 2: Persistently high problematic SMU, persistently average SMU frequency (ref. = class 4: persistently low problematic SMU, variably high SMU frequency)														
	Model A			Model B			Model C			Model D			Model E (Figure 4)		
	*B*	*SE*	*OR*	*B*	*SE*	*OR*	*B*	*SE*	*OR*	*B*	*SE*	*OR*	*B*	*SE*	*OR*
Controls															
Girl (ref. = boy)	−0.916***	.260	0.400	−1.050***	.271	0.350	−0.683*	.273	0.505	−0.393	.286	0.676	−0.500	.327	0.610
Pre‐vocational education (ref. = pre‐university)	0.447	.417	1.563	0.439	.438	1.552	0.432	.415	1.540	0.373	.476	1.457	0.294	.471	1.347
Intermediate education (ref. = pre‐university)	0.388	.424	1.474	0.327	.447	1.387	0.296	.431	1.345	0.419	.488	1.524	0.190	.474	1.210
Immigrant background (ref. = non‐immigrant)	0.608*	.272	1.837	0.682*	.287	1.979	0.701*	.287	2.017	0.473	.323	1.611	0.520	.333	1.691
Subjective well‐being															
Life satisfaction				−1.124**	.327	0.327							−0.793*	.321	0.454
Self‐esteem				−0.646	.350	0.526							0.309	.386	1.372
Self‐control															
Attention deficit							1.082**	.354	2.981				0.541	.429	1.752
Impulsivity							0.264	.367	1.308				−0.226	.424	0.805
Social competencies															
Perceived friendship competence										−2.885***	.414	0.057	−2.634***	.430	0.073

Abbreviations: *B*, logit coefficient; *OR*, odds ratio; Ref., reference category; *SE*, standard error; SMU, social media use.

* *p* < .05

** *p* < .01

*** *p* < .001.

**TABLE A3:** Results multinomial regression, membership class 3 (*n* = 1419)

	Class 3: Persistently low problematic SMU, persistently low SMU frequency (ref. = class 4: persistently low problematic SMU, variably high SMU frequency)														
	Model A			Model B			Model C			Model D			Model E (Figure 4)		
	*B*	*SE*	*OR*	*B*	*SE*	*OR*	*B*	*SE*	*OR*	*B*	*SE*	*OR*	*B*	*SE*	*OR*
Controls															
Girl (ref. = boy)	−1.098***	.188	0.333	−1.051***	.196	0.350	−1.360***	.196	0.257	−0.932***	.195	0.394	−1.147***	.212	0.318
Pre‐vocational education (ref. = pre‐university)	−0.581*	.242	0.560	−0.580*	.245	0.560	−0.571*	.254	0.565	−0.633*	.255	0.531	−0.633*	.288	0.532
Intermediate education (ref. = pre‐university)	−0.533*	.261	0.587	−0.595*	.266	0.552	−0.549*	.269	0.578	−0.561*	.273	0.570	−0.633*	.293	0.531
Immigrant background (ref. = non‐immigrant)	0.374	.219	1.453	0.368	.225	1.446	0.377	.232	1.459	0.331	.227	1.394	0.272	.253	1.315
Subjective well‐being															
Life satisfaction				−0.574*	.250	0.564							−0.468	.251	0.628
Self‐esteem				0.518	.269	1.681							0.881**	.294	2.423
Self‐control															
Attention deficit							0.162	.277	1.180				0.002	.307	1.006
Impulsivity							−1.776***	.386	0.172				−2.060***	.406	0.130
Social competencies															
Perceived friendship competence										−1.170***	.289	0.311	−1.606***	.352	0.202

Abbreviations: *B*, logit coefficient; *OR*, odds ratio; Ref., reference category; *SE*, standard error; SMU, social media use.

* *p* < .05

** *p* < .01

*** *p* < .001.

**TABLE A4:** Multinomial regression, membership class 1, 2, 3, and 4 (*n* = 1419)

	Class 1: Variably high problematic SMU, variably high SMU frequency			Class 2: Persistently high problematic SMU, persistently average SMU frequency			Class 3: Persistently low problematic SMU, persistently low SMU frequency			Class 4: Persistently low problematic SMU, variably high SMU frequency		
	Ref. = c2	Ref. = c3	Ref. = c4	Ref. = c1	Ref. = c3	Ref. = c4	Ref. = c1	Ref. = c2	Ref. = c4	Ref. = c1	Ref. = c2	Ref. = c3
Controls												
Girl (ref. = boy)	1.674***	2.321***	1.174***	−1.674***	0.647*	−0.500	−2.321***	−0.647*	−1.147***	−1.174***	0.500	1.147***
Pre‐vocational education (ref. = pre‐university)	1.150*	2.078***	1.445**	−1.150*	0.927*	0.294	−2.078***	−0.927*	−0.633*	−1.445**	−0.294	0.633*
Intermediate education (ref. = pre‐university)	0.309	1.132*	0.499	−0.309	0.823	0.190	−1.132*	−0.823	−0.633*	−0.499	−0.190	0.633*
Immigrant background (ref. = non‐immigrant)	−0.420	−0.172	0.100	0.420	0.248	0.520	0.172	−0.248	0.272	−0.100	−0.520	−0.272
Subjective well‐being												
Life satisfaction	−0.556	−0.881*	−1.349***	0.556	−0.325	−0.793*	0.881*	0.325	−0.468	1.349***	0.793*	0.468
Self‐esteem	0.330	−0.243	0.638	−0.330	−0.573	0.309	0.243	0.573	0.881**	−0.638	−0.309	−0.881**
Self‐control												
Attention deficit	0.509	1.048*	1.050**	−0.509	0.539	0.541	−1.048*	−0.539	0.002	−1.050**	−0.541	−0.002
Impulsivity	1.833***	3.667***	1.607***	−1.833***	1.834***	−0.226	−3.667***	−1.834***	−2.060***	−1.607***	0.226	2.060***
Social competencies												
Perceived friendship competence	2.424***	1.396**	−0.210	−2.424***	−1.028*	−2.634***	−1.396**	1.028*	−1.606***	0.210	2.634***	1.606***

Note

All estimates denote logit coefficients.

Abbreviations: c, class; Ref., reference category; SMU, social media use.

* *p* < .05

** *p* < .01

*** *p* < .001.
