# Supplementary material for: The course of problematic social media use in young adolescents: A latent class growth analysis
Source: Child Dev. 2021 Nov 15;93(2):e168–87. doi: 10.1111/cdev.13712 (PMC9298889; doi:10.1111/cdev.13712)
Supplement: Supplementary file 1 — Supplementary Material [file CDEV-93-e168-s001.docx]

**The course of problematic social media use in young adolescents: A latent class growth analysis**

# **Online Supplement**

| **Table S1. Difference in latent class growth model estimates by sample (*n* = 1,419)** | | | | |
| --- | --- | --- | --- | --- |
|  | **Problematic SMU** | | **SMU frequency** | |
| **Sample 1** | **Estimate** | ***SE*** | **Estimate** | ***SE*** |
| Intercept | -0.204** | 0.067 | 3.992*** | 0.067 |
| Slope | -0.031 | 0.033 | 0.149*** | 0.026 |
| Variance intercept | 0.729*** | 0.068 | 1.421*** | 0.090 |
| Variance slope | 0.029* | 0.015 | 0.013 | 0.015 |
| **Sample 2** | **Estimate** | ***SE*** | **Estimate** | ***SE*** |
| Intercept | -0.093 | 0.061 | 4.081*** | 0.065 |
| Slope | 0.027 | 0.033 | 0.169*** | 0.033 |
| Variance intercept | 0.780*** | 0.071 | 1.378*** | 0.096 |
| Variance slope | 0.014 | 0.022 | 0.014 | 0.023 |
| **Differences between samples 1 and 2** | **Estimate** | ***z*** | **Estimate** | ***z*** |
| Intercept | -0.111 | -1.222 | -0.090 | -0.968 |
| Slope | -0.058 | -1.249 | -0.020 | -0.487 |
| Variance intercept | -0.051 | -0.517 | 0.043 | 0.340 |
| Variance slope | 0.015 | 0.571 | -0.001 | -0.038 |
| *Notes.* SMU = social media use; SE = standard error; z = z-score. Sample 1 *n* = 799 (56.3%), sample 2 *n* = 620 (43.7%). Estimates for Problematic SMU were based on Poisson regression.  *** *p* < 0.001, ** *p* < 0.01, * *p* < 0.05. | | | | |

**Figure S1. BIC-model fit of the (zero-inflated) Poisson LCGMs with 1 to 6 classes, n = 1,419**

*Notes.* BIC = Bayesian Information Criterion (BIC). Latent class growth models (LCGMs) include the co-trajectories of problematic social media use and social media use frequency.

**Figure S2. Model specification of the parallel latent class growth model.**

*Notes.* Probl. SMU = problematic social media use; SMU freq. = social media use frequency; I = intercept; S = slope; Q = quadratic slope; C = latent class. Circles denote latent variables. Squares denote observed variables. Numbers indicate the values of the constrained factor loadings.
